# Genome-wide association studies dissect the G × E interaction for agronomic traits in a worldwide collection of safflowers (*Carthamus tinctorius* L.)

**DOI:** 10.1007/s11032-022-01295-8

**Published:** 2022-04-12

**Authors:** Huanhuan Zhao, Keith W. Savin, Yongjun Li, Edmond J. Breen, Pankaj Maharjan, Josquin F. Tibbits, Surya Kant, Matthew J. Hayden, Hans D. Daetwyler

**Affiliations:** 1 School of Applied Systems Biology, La Trobe University, Bundoora, VIC 3083 Australia; 2 Agriculture Victoria, AgriBio, Centre for AgriBioscience, Bundoora, VIC 3083 Australia; 3 Agriculture Victoria, Grains Innovation Park, Horsham, VIC 3400 Australia

**Keywords:** Safflower, Agronomic traits, G × E interaction, GWAS, Mixed linear model

## Abstract

Genome-wide association studies were conducted using a globally diverse safflower (*Carthamus tinctorius* L*.*) Genebank collection for grain yield (YP), days to flowering (DF), plant height (PH), 500 seed weight (SW), seed oil content (OL), and crude protein content (PR) in four environments (sites) that differed in water availability. Phenotypic variation was observed for all traits. YP exhibited low overall genetic correlations (*r*_Goverall_) across sites, while SW and OL had high *r*_Goverall_ and high pairwise genetic correlations (*r*_Gij_) across all pairwise sites. In total, 92 marker-trait associations (MTAs) were identified using three methods, single locus genome-wide association studies (GWAS) using a mixed linear model (MLM), the Bayesian multi-locus method (BayesR), and meta-GWAS. MTAs with large effects across all sites were detected for OL, SW, and PR, and MTAs specific for the different water stress sites were identified for all traits. Five MTAs were associated with multiple traits; 4 of 5 MTAs were variously associated with the three traits of SW, OL, and PR. This study provided insights into the phenotypic variability and genetic architecture of important safflower agronomic traits under different environments.

**Supplementary Information:**

The online version contains supplementary material available at 10.1007/s11032-022-01295-8.

## Introduction

Safflower (*Carthamus tinctorius* L*.*) is a member of the Compositae family, grown as a vegetable, cut flower, herbal medicine, animal feed, birdseed, and oilseed, etc. in over 60 geographical regions covering the Middle East, Africa, America, Europe, and Asia (Knowles and Ashri 1995). In recent years, with a growing demand for healthy cooking oil and clean biofuel and bio-lubricants, safflower has emerged as a modern industrial oilseed crop due to its higher oleic and linoleic acid content compared to other oilseed crops (Fernández-Martinez et al. 1993; Khalid et al. 2017). In 2019, FAO data showed safflower seed production world-wide was approximately 0.6 million tonnes, and the top 4 largest growers (Kazakhstan, United States, Russian Federation, and Mexico) produce over 75% of total production (FAO 2019). The Australian safflower growing area is currently about 40,000 ha, down from its peak of 74,688 ha in 1979 (Jochinke et al. 2008). As a potential crop that could grow in a drier environment, safflower is gaining more research attention (Li and Mündel 1996).

To date, genetic analyses for agronomic traits in safflower have largely been undertaken using conventional family-based methods (Kotecha 1979; Ramachandram and Goud 1981). This has allowed the identification of genes and quantitative trait loci (QTL) for traits such as plant height, seed oil content, and days to flowering (Hamdan et al. 2012; Pearl et al. 2014). Association mapping approaches have also been used to identify QTL in safflower. Study using AFLP markers detected four marker-trait associations (MTAs) for PH under drought conditions in safflower (Ebrahimi et al. 2017a). Six MTAs for PH, five MTAs for DF, and several MTAs for oil content, oleic acid content, and linoleic acid content were identified in an association study using microsatellite markers (Ambreen et al. 2018). The *Fad2* gene family (Fatty acid desaturases, FAD) in safflower has been sequenced with genes being isolated and cloned (Cao et al. 2013; Wood et al. 2018). However, no genome-wide association studies (GWAS) based on single nucleotide polymorphisms (SNPs) markers have been reported in safflower.

Statistical methods used in GWAS analysis are important for identifying MTAs for complex traits (Wang et al. 2019; Zhang et al. 2010). Single-locus GWAS with mixed linear models (MLM-GWAS) has been widely used to detect the MTAs for agronomic traits in a variety of plants, including wheat (Ledesma-Ramírez et al. 2019), rapeseed (Qu et al. 2017), soybean (Leamy et al. 2017a), etc.. To increase power to discover SNP with small effects and reduce false-positive associations, summary statistic-based methods (meta-GWAS) have been adopted in some studies (Joukhadar et al. 2021; Pasaniuc and Price 2017). In canola, a meta-GWAS analysis identified 79 genomic regions conferring potential candidate resistance to canola blackleg disease, more significant SNPs than single-locus GWAS (Fikere et al. 2020). Differing from single-locus MLM-GWAS testing one marker at a time, multi-locus GWAS have been applied by fitting all loci simultaneously to improve fine-mapping (Kaler et al. 2020; Tamba et al. 2017). As a multi-locus Bayesian method, BayesR simultaneously accommodates all SNPs in the model, and SNPs effects were a mixture of four normal distributions, which include SNPs with 0, small, and moderate effects. In each distribution, fewer SNPs explain the gradually more genetic variance (Daetwyler et al. 2014; Erbe et al. 2012). BayesR has been used to identify QTL or associations in dairy cattle and wheat (Pasam et al. 2017; Xiang et al. 2021).

The variation in phenotypes among genotypes in different environments is evaluated as the extent of the genotype-by-environment interaction (G × E), which is also referred to as the traits phenotypic plasticity (Bradshaw 1965). Identifying G × E interaction patterns and their genetic basis under multi-environment trials can deepen the knowledge of the genetic architecture of traits (Das et al. 2019; Kusmec et al. 2018). In a canola study, 12 environment-stable QTL and 43 environment-specific QTL were detected for flowering time in three different ecological conditions, which provided new insights into the genetic regulatory network underlying the control of flowering time (Li et al. 2018a). Few studies investigating G × E interaction patterns have been reported in safflower, which were carried out to evaluate genotypes and yield stability (Alizadeh et al. 2017; Jamshidmoghaddam and Pourdad 2013).

In Australia, crop production is challenged by spatial drought patterns due to seasonal rainfall and high temperatures (Chenu et al. 2013). Therefore, understanding the G × E interaction and genetic basis underlying grain yield and related agronomic traits are important for safflower breeding. In this study, a globally diverse Genebank collection of 406 accessions was grown in 4 different field environments (2 trials in one location but with different field management in 2017 and 2 locations in 2018). The aims were to (1) assess genetic variability in the different environments and the level of G × E interaction for grain yield and related agronomic traits and (2) identify MTAs via GWAS at each environment to study the genetic basis of the G × E interaction for grain yield and related agronomic traits.

## Materials and methods

### Plant material and phenotyping

A total of 406 globally diverse safflower accessions were sourced from the Australian Grain Genebank (AGG), including elite cultivars, breeding lines, and landraces. Accessions information and the field trial experiment design are previously described (Zhao et al. 2021). In brief, with a randomized complete block design, all accessions were sown at two field sites in two consecutive years (2017 and 2018, a total of four sites) with plot size of 1 m x 5 m, 5 rows in each plot, and 220 seeds sowed per plot. Sites 1 and 2 were sown in 2017 at the same location (Horsham, Victoria) in a low rainfall zone. Site 1 was flood irrigated before sowing and considered an optimal site with a full soil water profile. Site 2 was rainfed, with soil water stress starting in late spring (during the flowering stage). Sites 3 and 4 were sown in 2018. Site 3 was at the same location as sites 1 and 2 but was rainfed and experienced soil water stress during the entire growing season, with minimal rain in the early spring and high temperature towards the end of the season. Site 4 was in a higher rainfall zone (Wonwondah, Victoria) and received more rain overall than site 3, but also experienced soil water stress.

Days to flowering (DF) was recorded as the number of days from sowing to 25% of the plot flowering. Plant height (PH) was measured at the late flowering stage from the ground surface to the top of the plot canopy in cm. Seed weight (SW) was measured as random 500 achenes from the whole plot in grams. Grain yield was measured as yield per plot (YP) in kilograms harvested by machine. Seed protein (PR) and seed oil content (OL) were determined by near-infrared reflectance spectroscopy (NIR, Foss Pacific Pty Ltd, Denmark) with calibration by the Dumas nitrogen combustion method for protein (TruMac, Leco Corporation St Joseph USA), and the Soxhlet extraction for oil (Soxtec 25,050, FOSS, Hilleroed, Denmark). The NIR prediction models R-squared (*R*^*2*^) and standard error of prediction (SEP) were 0.93 and 0.7% for seed protein content and 0.95 and 1.2% for seed total oil content.

### Statistical analysis of phenotypic data

Summary statistics were calculated for each trait at each site. The best linear unbiased estimates (BLUE) for each trait at each site were calculated by a single site linear mixed model with safflower accessions fitted as fixed effects. The model was illustrated as:

1 $${Y}_{mijk}=\mu +{g}_{m}+{R}_{j}+{r}_{j}+{c}_{k}+{\varepsilon }_{mijk}$$

where *Y*_*mijk*_ is the phenotypes of accessions *m* in rep *j* at row *i*, column *k*; *µ* is the overall mean, *g*_*m*_ is the fixed accession genetic effect, and *R*_*j*_ is the replicate effect; *r*_*i*_ is the row effect, *c*_*k*_ is the column effect, and *ɛ*_*mijk*_ is the residual, including the AR1 × AR1 covariance structure to adjust spatial variation.

Pearson’s correlation at each site was calculated based on the BLUEs of each trait. BLUEs were used as the “phenotypes” for the GWAS.

To assess the G × E level for each trait, the four sites were combined, and the genetic effect associated with accessions was decomposed into two components, the genetic effect of accessions and the interaction effect between accessions and sites (G × E effect), which were assumed to be homogenous for all sites. The linear mixed model was:

2 $${Y}_{ijk}=\mu +{S}_{i}+{R}_{j}+{G}_{k}+{SG}_{ik}+{\varepsilon }_{ijk}$$

where *Y*_*ijk*_ is the phenotype of accession *k* in rep *j* at site *i*, *µ* is the overall mean, *S*_*i*_ is the fixed *i*-th site effect, *R*_*j*_ is the fixed replicate effect, *G*_*k*_ is the random accession genetic effect, *SG*_*ik*_ is the random G × E effect, and ɛ_*ijk*_ is the residual. Two models, including and excluding the G × E effect, were compared, and a log-likelihood ratio test was used to test the significance of the G × E effect for each trait (Kendall and Stuart 1979). The genetic correlation among the four sites (*r*_Goverall_) was estimated as the ratio of the genetic effect of accessions to total genetic variance, calculated as *r*_Goverall_ = ${\sigma }_{G}^{2}/({\sigma }_{G}^{2}+{\sigma }_{GE}^{2})$, where ${\sigma }_{G}^{2}$ is the genetic variance of accessions and ${\sigma }_{GE}^{2}$ is the variance for G × E interaction. High genetic correlation among sites indicated low G × E interaction, while low genetic correlation indicated high G × E interaction (Li et al. 2016).

A heterogeneous variance structure was also fitted in the linear mixed model, which assumes that accessions genetic effect is independent at each site. It can be illustrated as:

3 $${Y}_{ijk}=\mu +{S}_{i}+{SR}_{ij}+{SG}_{ik}+{\varepsilon }_{ijk}$$

where the terms are the same as above, with the site as a fixed effect and the accession and trial replicate effects both nested within sites as a random effect with different variance for each site. The residual variance was also nested within the site, with the AR1 × AR1 covariance structure used to adjust spatial variation across columns and rows. The genetic correlation of the accession effect between two sites was calculated as *r*_Gij_ = ${\sigma }_{GiGj}/\sqrt{{\sigma }_{Gi}^{2}{*\sigma }_{Gj}^{2}}$, where the ${\sigma }_{Gi }^{2}$ and ${\sigma }_{Gj }^{2}$ are the variance of the accession genetic effect at sites i and j, respectively. The ${\sigma }_{GiGj}$ is the covariance of the accessions genetic effect at sites *i* and *j*. Similar to the above, high genetic correlations between two sites indicated a low G × E interaction. The significance of the genetic correlation between two sites was tested for deviation from 1 using likelihood ratio tests. If *r*_Gij_ significantly differed from 1, it suggested the ranking of accessions at the two sites was different. Akaike information criterion (AIC) was used to compare the fitness of models 2 and 3.

### SNP genotyping and population structure

A total of 349 accessions were genotyped using a genotyping-by-sequencing assay as described in (Zhao et al. 2021). In brief, genomic DNA was extracted from six crushed seeds per accession, digested with restriction endonucleases PstI (6-bp cutter) and MseI (4-bp cutter), followed by the amplification, purification, and sequencing by Illumina Hiseq 3000 sequencer. SNP discovery and genotype calling were conducted with custom scripts, and SNPs were filtered for a missing data rate < 30% and minor allele frequency (MAF) > 0.01 and imputed with LinkImpute (Money et al. 2015). A total of 318 samples were passed the filtering, and population structure was evaluated from the genomic relationship matrix (GRM) according to VanRaden (VanRaden 2008). SNPs were further filtered with MAF > 0.05 and heterozygosity < 0.3 for 318 samples for the genome-wide association study. The physical position of the filtered SNP was determined by mapping their flanking sequences to a draft safflower genome assembly (unpublished data) with 12 main scaffolds (pseudochromosomes). Linkage disequilibrium (LD) was calculated for all pairwise SNP using PLINK (Purcell et al. 2007).

### Genome-wide association study

Single site GWAS was conducted for each trait using the BLUEs of each trait as the “phenotypes” (Supplementary Table s1). First, a single SNP regression model, referred to as MLM-GWAS, implemented in the GCTA software (Yang et al. 2011), was performed with the GRM fitted to account for population structure. Second, the Bayesian multi-locus approach-BayesR was performed using the Markov chain Monte Carlo (MCMC) method with 50,000 iterations and 25,000 burn-in. SNPs with large effects were declared if they had a nonzero effect with at least a 0.7 posterior probability, averaged over 5 runs (Erbe et al. 2012). And third, meta-GWAS implemented in the software Metal (Willer et al. 2010) was performed for each trait, with each single site MLM-GWAS treated as an independent study. Manhattan and quantile–quantile (Q-Q) plot generated with an R script (Yu et al. 2006) were used to visualize associations for each trait. SNP identified by all three methods were considered candidate MTAs for each trait.

## Results

### Phenotypic variation and correlations

**Fig. 1:**
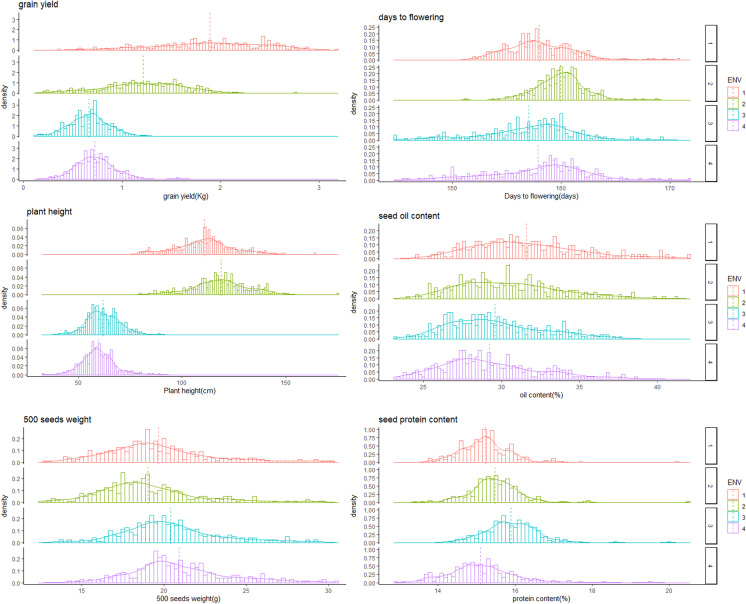
Distribution of grain yield (YP), plant height (PH), days to flowering (DF), 500 seed weight (SW), seed protein (PR), and oil content (OL) among the 406 safflower accessions at each of the four field trial sites (ENV, dashed line shows the trait mean)

In total, 406 globally diverse safflower accessions were evaluated in four field trials. The phenotypic distributions and means for grain yield (YP), plant height (PH), days to flowering (DF), 500 seed weight (SW), seed protein (PR), and oil content (OL) are shown in Fig. 1. The mean YP was the highest at site 1 (1.89 kg/plot), a third less at site 2 (1.21 kg/plot), and halved at sites 3 and 4 (0.66 and 0.72 kg/plot) (Supplementary Table s2). The distribution for YP was much narrower at sites 3 and 4 compared with sites 1 and 2 for YP (Fig. 1). PH showed a similar distribution pattern to YP, with lower means at sites 3 and 4 (~ 60 cm) compared to sites 1 and 2 (~ 115 cm). DF had the highest mean value and narrowest distribution at site 2 (~ 160 days, Fig. 1). There were differences in trait means between sites for the three seed traits (SW, PR, and OL), but they were more subtle than those observed for YP and PH. The SW mean was higher at sites 3 and 4 (~ 20.6 g) than at sites 1 and 2 (19.56 g and 18.77 g, respectively). The mean of PR ranged from 15.14 to 15.92% across four sites, and OL decreased about 1% with different water stress environments, from 31.83% at site 1 down to 29.69% at site 3. Similarly, the distributions for the three seed traits did not change dramatically across the four sites (Fig. 1, Supplementary Table s2).

Pearson correlations between traits at each site showed that YP was positively correlated with PH (0.34–0.479) and negatively correlated with PR (− 0.208– − 0.405). SW was negatively correlated with OL (− 0.518– − 0.505), PR (− 0.55– − 0.383), and PR was positively correlated with OL (0.19–0.639) over all sites. However, DF is positively correlated with PH at sites 1, 3, and 4 (0.464–0.62) and negatively correlated with PH at site 2 (Table 1).

**Table 1:** Pearson correlation between traits at each site

Site	Trait	OL	PH	PR	SW	YP
	DF	0.101	0.464	− 0.093	− 0.215	0.101
ENV1	OL		− 0.109	0.19	− 0.508	0.154
	PH			− 0.222	0.01	0.441
	PR				− 0.383	− 0.369
	SW					0.034
	DF	0.29	− 0.076	0.2	− 0.352	− 0.156
ENV2	OL		− 0.075	0.203	− 0.505	0.03
	PH			− 0.317	0.129	0.479
	PR				− 0.484	− 0.405
	SW					0.206
	DF	0.027	0.579	0.074	− 0.17	0.097
ENV3	OL		− 0.058	0.513	− 0.51	− 0.029
	PH			− 0.078	− 0.051	0.42
	PR				− 0.55	− 0.29
	SW					0.177
	DF	0.02	0.62	− 0.02	− 0.152	0.192
ENV4	OL		− 0.112	0.639	− 0.518	0.104
	PH			− 0.109	− 0.001	0.34
	PR				− 0.471	− 0.208
	SW					0.07

### G × E interaction

G × E interactions for each trait were determined through combined site analysis, and the overall genetic correlations ranged from 0 to 1 among sites for all traits (model 2). The model including G × E interaction effects had a higher log-likelihood than the model excluding G × E interactions, and the G × E interaction effects were all significant (Supplementary Table s3, s4). High overall genetic correlations (*r*_Goverall_) among sites were observed for SW and OL (0.95 and 0.94, respectively), indicating low G × E interaction for those traits, while low r_Goverall_ for YP (0.48) indicated a strong G × E interaction. G × E levels were moderate for PH, DF, and PR, with r_Goverall_ value ranging from 0.67 to ~ 0.79 (Supplementary Table s4).

To account for differences in G × E interactions between sites, a linear mixed model assuming heterogeneous genetic and residual variances (model 3) was adopted. The AIC was lower for all traits for model 3 than for model 2, suggesting that model 3 fitted the data better (Supplementary Table s5). The genetic correlation (*r*_Gij_) between pairwise site combinations varied; however, traits with high *r*_Goverall_ also had high r_Gij_ between pairwise sites (Table 2). According to Robertson (Robertson 1959), a correlation of performance between environments ≤ 0.8 indicates a considerable re-ranking of individuals. SW and OL had high pairwise genetic correlations between all sites with *r*_Gij_ values > 0.9, especially between sites 3 and 4, in which a *r*_Gij_ (0.99) was not significantly different from 1. As the rest traits all had *r*_Gij_ ≥ 0.80 between sites 3 and 4, suggesting that those two sites could be treated as a single site with limited re-ranking. Genetic correlations for PH were uniformly high between pairwise sites (*r*_Gij_ ≥ 0.80), while the genetic correlations for DF and YP varied, with those for YP being the most variable.

**Table 2:** Genetic correlations (*r*_Gij_, with standard error in parenthesis) between sites for six traits were calculated with model 3

Site pair		Trait					
ENV_i	ENV_j	YP	PH	DF	SW	OL	PR
1	2	0.69 (0.04)	0.84 (0.02)	0.6 (0.04)	0.96 (0.01)	0.94 (0.01)	0.89 (0.02)
1	3	0.45 (0.06)	0.81 (0.05)	0.85 (0.02)	0.93 (0.01)	0.95 (0.01)	0.7 (0.04)
1	4	0.68 (0.05)	0.81 (0.03)	0.85 (0.02)	0.93 (0.01)	0.95 (0.01)	0.67 (0.04)
2	3	0.39 (0.07)	0.87 (0.04)	0.35 (0.05)	0.93 (0.01)	0.9 (0.01)	0.78 (0.03)
2	4	0.46 (0.06)	0.8 (0.03)	0.36 (0.05)	0.92 (0.01)	0.91 (0.01)	0.73 (0.04)
3	4	0.82 (0.06)	0.87 (0.05)	0.99 (0)*	0.99 (0)*	0.99 (0)*	0.94 (0.02)

*Genetic correlation is not significantly differed from value of 1 at 0.05 level

### Genome-wide associations

The heatmap of the genomic relationship matrix (**G**) revealed a strong population structure among 318 accessions (Supplementary Figure s1), which was consistent with the previous observation (Zhao et al. 2021). After further filtering, a total of 1806 SNPs were used for GWAS studies, with 1780 positioned on the 12 pseudochromosomes of the draft safflower genome assembly, about 100–200 SNPs per pseudochromosome. LD decayed rapidly over a short physical distance, followed by a slower decline over longer pairwise distances (Supplementary Figure s2).

**Fig. 2:**
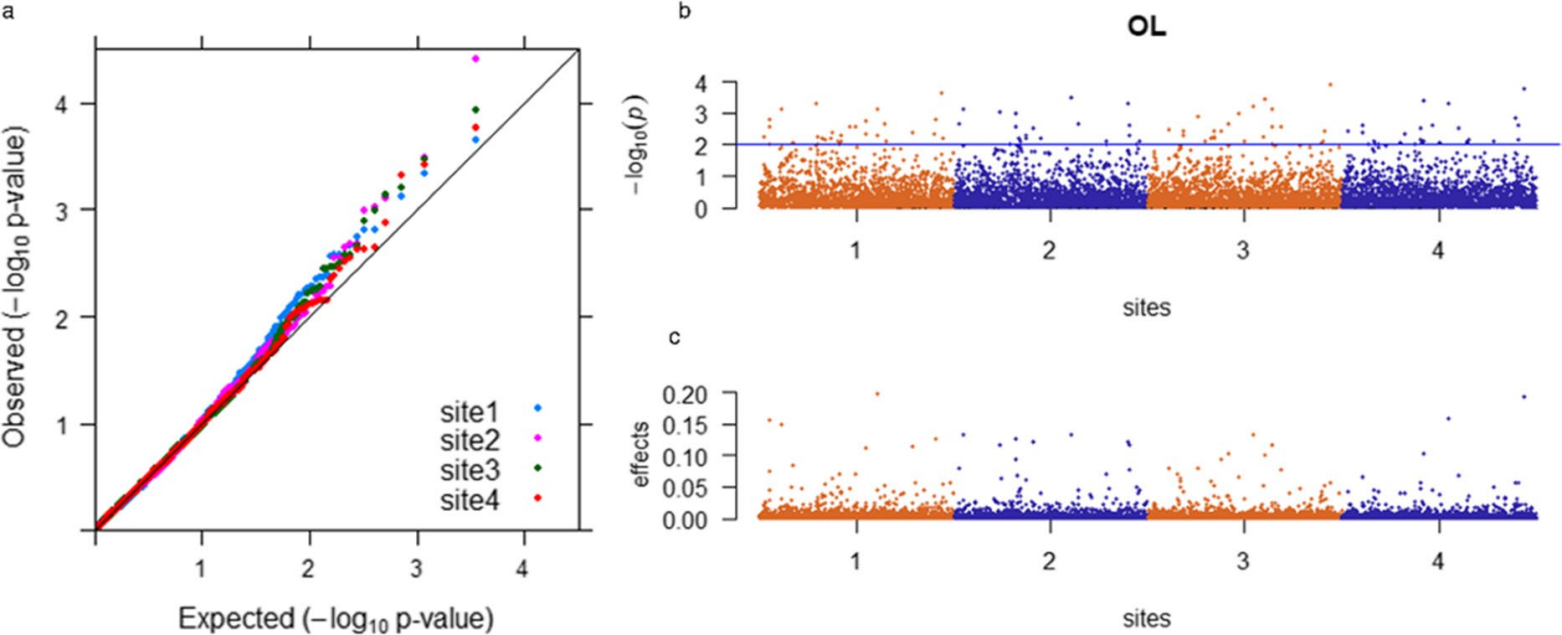
**a** QQ plot for seed oil content (OL) at the four trial sites; **b** negative log_10_ *p* values are plotted against for all SNPs across four sites for OL, and the blue line indicates the threshold of significance with the MLM-GWAS method; and **c** SNP effects are plotted against for all SNPs across four sites for OL with the BayesR GWAS method. SNPs are sorted according to physical position in the 12 pseudochromosomes and are in the same order for each of the four trial sites

Combined QQ plots for the four sites showed that the inclusion of **G** matrix in the GWAS effectively accounted for the observed population structure (Fig. 2, Supplementary Figure s3). A relaxed significance threshold of -log10(p) ≥ 2 was used to denote MTAs in the MLM-GWAS, which resulted in the identification of between 41 and 71 putative MTAs for each trait across the four sites. For the meta-GWAS, the number of significant SNPs was more than twice the number found in the MLM-GWAS for OL, SW, and PR. Fewer MTAs were detected using the BayesR method, especially for PR (Table 3). SNPs with large effects in the BayesR analysis typically overlapped with SNPs above the significance threshold in the MLM-GWAS (Fig. 2, Supplementary Figure s3).

**Table 3:** Number of MTAs identified by different GWAS methods

Trait	MLM-GWAS	Meta-GWAS	BayesR	Common MTAs	Shared MTAs (> = 2 sites)	Site-specific MTAs
DF	52	62	38	16	2	14
OL	54	116	38	30	12	18
PH	71	71	32	17	7	10
PR	55	119	5	5	3	2
SW	41	150	27	20	11	9
YP	59	48	10	4	1	3

A total of 92 significant MTAs were detected by all three GWAS approaches (Table 3, Supplementary Tables s6-s11). Heatmap of pairwise LD between significant MTAs for each trait was plotted to show that MTAs were not tightly linked (Supplementary Figure s4). By comparing MLM-GWAS and BayesR results, the significant MTAs were classified as site-specific or shared among sites, where sites 3 and 4 were treated as a single site. Traits with low overall G × E (high genetic correlation) had a higher percentage of shared MTAs across sites (SW and OL), while traits with high to moderate overall G × E (PH, DF, and YP) detected limited shared MTA between pairwise sites. Five and six MTAs shared across all sites were observed for SW and OL, respectively, with Locus 6195 and 28,935 for OL and Locus 3057 and 27,064 for SW showing large effects (> 0.15). PR had three shared MTAs, and locus 3057 with large effects were detected across all sites. Seven MTAs for PH were shared between two sites, and five of seven were between sites 1 and 2. Two and one MTAs were shared between two sites for DF and YP, respectively. All site-specific MTAs were consistent in effect direction but varied in magnitude across sites, except the MTAs for locus 25,179 and 6025 associated with DF and PH, respectively, which had opposite directions with near zero effect at nonsignificant sites. The number of site-specific MTAs observed for each trait differed among sites. 12 out of 18 site-specific SNPs for OL were observed at site 3 (or site 4), six of nine site-specific MTAs for SW were observed at site 1, and eight of 14 site-specific MTAs for DF were observed at site 1 (Table 3, Supplementary Tables s6–11).

Five MTAs had significant associations with more than one trait. Locus 5628 was associated with PH at sites 2 and 3 and DF at site 1. Locus 9819 was associated with DF at site 1, OL at site 3, and PR at sites 2 and 3. Locus 17,302 and 3057 increased SW but decreased PR and OL at site 2 and site 3 (or site 4), respectively. Locus 28,935 was associated with low SW and high OL at sites 2 and 3 (or site 4) (Table 4).

**Table 4:** MTAs with effects on multiple traits with their MAF, physical position, Z-score, and *p* value from the meta-GWAS

SNP	MAF	Pseudochromosome	Physical position	Trait	Site	Z-score	*P* value
Locus5628	0.39	1	83,711,358	DF	1	4.167	3.08E-05
				PH	2,3	4.638	3.51E-06
Locus9819	0.1	8	79,766,528	DF	1	4.598	4.26E-06
				PR	2,3,4	5.709	1.14E-08
				OL	3	4.763	1.91E-06
Locus17302	0.1	12	1,089,790	PR	2	-4.555	5.24E-06
				SW	1,2	4.97	6.68E-07
				OL	2	-3.701	0.000215
Locus3057	0.06	7	15,110,222	PR	1,2,3,4	-8.97	2.95E-19
				SW	1,2,3,4	7.021	2.2E-12
				OL	3,4	-4.45	8.58E-06
Locus28935	0.08	8	23,744,954	SW	2,3,4	-5.623	1.88E-08
				OL	1,2,3,4	6.767	1.32E-11

## Discussion

Understanding G × E is an important initial step to developing strategies for a breeding program in the target environment(s). Our results showed that G × E patterns differed between safflower traits. The identification of site-shared and site-specific MTAs in GWAS provides knowledge to broaden our understanding of the genetic basis of G × E interactions for important safflower traits.

### Different G × E interaction patterns were observed for safflower traits

The seasonal rainfall in the Victoria Wimmera region (Horsham and Wonwondah) had an impact on safflower agronomic traits. Safflower is normally sown in Winter in Australia to maximize the usage of the available water from Winter and early Spring rain (Wachsmann et al. 2008). In our study, we observed that water stress during flowering decreased safflower grain yield substantially. Further, insufficient Spring rain heavily reduced safflower production via poor biomass accumulation. Similar grain yield instability induced by rainfall patterns has also been reported in other crops (Sadras and Dreccer 2015). Besides grain yield, a 1% decrease of OL was observed under differing water stress, consistent with previous studies that oil content decreased under drought stresses (Ebrahimi et al. 2017b; Joshan et al. 2019). The positive correlation between OL and PR (0.14 to ~ 0.46 across sites), which was also reported in a previous study (*r* = 0.476) (Oz 2016), indicated that artificial selection for OL in safflower has a limited impact on seed protein compared with soybean (Leamy et al. 2017b). The negative correlation between SW with PR and OL across all four sites suggests a negative relationship between carbohydrate accumulation and protein and oil accumulation, which could be similar to the competition in cereal crops (Bjarnason and Vasal 1992; Pasam et al. 2012).

The overall G × E interactions were significant for all the traits studied. However, there were different levels of G × E for the different traits. The heterogeneous model further revealed detailed G × E patterns for each trait, which indicated the rank changes of accessions between sites. The high G × E observed for YP across sites was consistent with studies in other crops (He et al. 2019; Tolessa et al. 2013). Low to moderate pairwise genetic correlation indicated re-ranking for YP was high among sites. Only 4 accessions showed yield stability through presence in the top 50 high yield accessions across all sites, which could be used for the future breeding program. The cultivars and breeding lines performed well at site 1 (19 accessions out of the 50 top YP accessions) but not at the other three sites, suggesting that introgression of water stress tolerance from landraces could improve safflower yield stability. The low level of G × E for OL with limited re-ranking across sites observed in our study was also indicated in a soybean study (Sudarić et al. 2006). According to the BLUEs for each site, about half of the top 30 accessions with high OL were cultivars and breeding lines, reflecting breeding efforts to improve OL in safflower cultivars. Although a moderate *r*_Goverall_ was observed for DF and PH, the pairwise genetic correlation showed that lines were reranked strongly for DF at site 2 compared with other sites. The genetic divergence of DF among the accessions in response to water stress at flowering implied DF is important in developing drought tolerant varieties (Bhandari et al. 2020).

### GWAS identified MTAs for safflower traits

GWAS has been widely used to study the genetic basis of the important agronomy traits with diverse germplasm in crops (Liu and Yan 2019). Multi-environment trials normally were combined to present the overall phenotypic variation for GWAS to detecting the associations between markers and traits (Landers and Stapleton 2014; Leamy et al. 2017c). However, with diverse germplasm, the phenotypic variation displayed under differed environments can be used to measure the plasticity of the traits or trait G × E level with proper statistical models (Des Marais et al. 2013; Malosetti et al. 2013). Environmental stable and environmental-specific MTAs can help our understanding of the genetic basis of trait G × E, and it also will enrich our knowledge of the genetic architecture of the important agronomy traits (Li et al. 2018b; Timpson et al. 2018). In our study, GWAS was carried out with a globally diverse safflower collection for six agronomic traits in four field trials that differed with water availability. MTAs shared across sites were identified for traits with low G × E, and site-specific MTAs were discovered for all traits with more site-specific MTAs than shared MTAs identified for moderate overall G × E traits.

A high number of significant MTAs were identified for seed oil content (OL) by all three GWAS approaches, of which 18 were shared across sites, and 12 were site-specific, indicating the complex genetic control of this trait. Studies with canola showed that 24 candidate genes were involved in fatty acid biosynthesis (Qu et al. 2017). In safflower, a transcriptome study showed that a significant number of transcription factors were involved in oil accumulation in safflower seeds (Li et al. 2021). The six MTAs shared across four sites will be of interest to safflower breeders and geneticists as sources of genetic variation to improve the seed oil content in safflower under different growing conditions. Similarly, numerous MTA (total 20) were identified for seed weight (SW), of which 11 were shared across sites. Three MTAs explaining more than 10–20% phenotypic variance across sites will provide useful information for breeders to modify SW in safflower (Supplementary Table s9).

The molecular basis for G × E interactions could be due to site-specific QTLs, gene expression, or differences in the magnitude of expression across environments (Des Marais et al. 2013; Li et al. 2018b). In our study, all site-specific MTAs showed differing allelic effects across sites for each trait (Supplementary Table s6–11); however, the effects were significant in some environments but not in other environments. We observed moderate overall G × E for PH and DF with a higher number of site-specific MTAs. Markers associated with PH and DF under drought conditions in safflower have been reported (Ebrahimi et al. 2017a). Only one MTA was identified for DF at site 2, which could be related to the narrow phenotypic variation observed at site 2 (Stich and Melchinger 2010). Few MTAs for PR and YP were detected by all three GWAS methodologies. However, those that were identified explained a high proportion of the phenotypic variation for each trait, indicating their potential importance for genetic improvement.

Correlations between traits can be caused by pleiotropy or a close linkage of loci associated with the traits (Chen and Lübberstedt 2010). Shared major genes or QTL for flowering time and plant height have been reported in soybean (Cober and Morrison 2010; Fang et al. 2017). In our study, locus 5628 was associated with DF at site 1 and PH at site 2 and 3, suggesting the MTA is likely tightly linked to different QTL affecting both traits, rather than being a single QTL with pleiotropic effects. In canola, a QTL affecting both OL and PR in repulsion was reported, suggesting the PR and OL biosynthesis pathways interfere and/or compete with one another (Chao et al. 2017). In maize, high OL and high PR were achieved using the *opaque*2 modifier genes. However, a yield reduction was noted (Vanous et al. 2019). In our study, we identified four MTAs affecting three traits, one MTA influencing both SW and OL, one MTA associated with PR and OL, and two MTAs interfering SW, OL, and PR. The allelic effects of those MTAs were consistent with the correlation observed in the field among the three traits that PR and OL are positively correlated, and both traits are negatively correlated with SW. This suggested that safflower breeding for PR and OL may differ from canola and maize. However, balancing seed weight and seed quality (OL and PR) would be a challenge. There were other strong phenotypic correlations, such as YP with PH and YP with PR, but associated markers were not identified. The reason could be the low number of significant SNPs that were observed for grain yield.

### The interplay of GWAS results and genetic architectures

SW and OL are known as highly heritable traits in many crops, while yield is more quantitative in nature (Ward et al. 2019; Xiao et al. 2019). The number of MTAs identified by the three GWAS methods did not fully reflect the complexity of the trait genetic architecture. One reason for this could be the thresholds used by the three methods. The *p* value used in our single locus MLM-GWAS was relaxed, and a significant number of candidate MTAs were observed for all traits. With meta-GWAS, we reported significant SNPs number with -log_10_P value at 3 instead of 2, which detected more significant SNPs for each trait indicating the increased power (Supplementary Table s12). However, multi-locus BayesR, which can improve association mapping resolution by removing multiple SNPs being in LD with the same QTL, could detect SNPs with larger effects (Kemper et al. 2015; Pasam et al. 2017). We observed fewer MTAs for all traits with the BayesR methods with the arbitrary threshold of 0.7 posterior probability of a SNP having an effect. This threshold may have been too stringent for polygenic traits such as YP and PR. Only 10 MTAs associated with YP, and 5 MTAs associated with PR were detected with BayesR, which explained 5–28% of the phenotypic variance.

### Heterogenous model fit the data better

Mixed linear models are widely used for G × E analysis in crop research (Smith et al. 2005). Falconer and Mackay (1996) suggested that the same trait measured in different environments should be considered as different (but correlated) traits. In our study, the homogenous model 2 combined the four sites together and estimated the overall GXE pattern with only three parameters. However, the heterogeneous model 3 treated each site as an independent environment, and a total of 26 parameters were estimated. The increased number of parameters allowed dissection of G × E among individual environments to reveal hidden patterns of genetic correlation between sites. Furthermore, the AIC, BIC, and Logl were improved for all traits, indicating model 3 was better able to fit the data (Hirotugu. 1998). These findings agreed with Malosetti et al. (2013), who compared different models to study G × E interactions and concluded that sophisticated mixed models are necessary to allow for heterogeneity of genetic variances and correlations across environments.

In conclusion, two mixed linear models were applied to analyse the G × E pattern for grain yield (YP), days to flowering (DF), plant height (PH), 500 seed weight (SW), seed oil content (OL), and seed protein content (PR) in a globally diverse safflower collection grown in four field trials. The heterogenous mixed linear model (MLM) fitted data better and provided a detailed estimation of the G × E pattern. We observed that different water stress conditions impacted the performance of each of these traits differently, with low overall G × E observed for OL and SW and high overall G × E for YP. In total, 92 MTAs were identified with large effects MTAs detected for OL, SW, and PR across all sites. Site-specific MTAs were detected for all traits with differed allelic effects, suggesting these MTAs could be associated with trait G × E. Five MTAs were associated with multiple traits. The uniform GWAS thresholds used in the study could have impacted the number of significant SNP identified for complex traits. This study has provided new insights into the genetic architecture of the traits studied, and it presents opportunities to exploit the MTA identified in breeding programs to increase yield stability and local adaptation in safflower.

## Supplementary Information

Below is the link to the electronic supplementary material.

Supplementary file1 (DOCX 973 KB)

Supplementary file2 (XLSX 121 KB)

## Data Availability

The phenotypic datasets supporting the conclusions of this article are included within the article and the attached additional files. And the genotype dataset, please see the previously published paper: Genomic prediction and genomic heritability of yield-related traits in Safflower https://doi.org/10.1002/tpg2.20064.
