# Supplementary material for: Genome-wide association studies dissect the G × E interaction for agronomic traits in a worldwide collection of safflowers (Carthamus tinctorius L.)
Source: Mol Breed. 2022 Apr 12;42(4):24. doi: 10.1007/s11032-022-01295-8 (PMC10248593; doi:10.1007/s11032-022-01295-8)

**Genome-wide association studies dissect the GXE interaction for agronomic traits in a world-wide collection of Safflowers** **(*Carthamus tinctorius* L.)**

Huanhuan Zhao^1,2*^, Keith W. Savin^2^, Yongjun Li^2^, Ed J Breen^2^, Pankaj Maharjan^3^, Josquin F. Tibbits ^2^, Surya Kant^1,3^, Matthew J. Hayden^1,2^, Hans D. Daetwyler^1,2^

**Supplementary Figure s1.** Heatmap of genomic relationships among 318 safflower accessions (subset from the previous publication, Zhao et al. 2021). Blue indicates less related, while orange means more related.


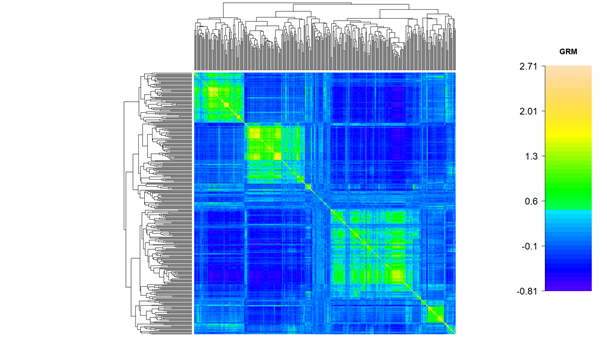


**Supplementary Figure s2.** Scatter plot of pairwise LD (r2) decay between SNPs positioned in the same pseudochromosomes with physical distance between SNPs (kilobases) for each locus (grey dots) and the average for each set of SNPs within 200kb distance (black dots).


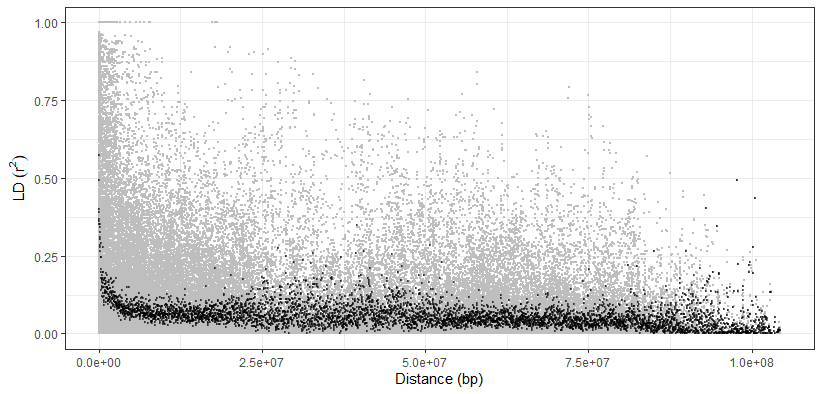


**Supplementary Fig s3.** Left, QQ plot for PR, SW, YP, DF and PH at the four trial sites. Right, negative Log_10_ p-values plotted against all SNP across the four sites. Blue line indicates the threshold of significance for MLM-GWAS method (upper), and SNP effects for the traits in the BayesR method (lower). SNPs are sorted according to physical position in 12 pseudochromosomes and are in the same order for each of the four trial sites.


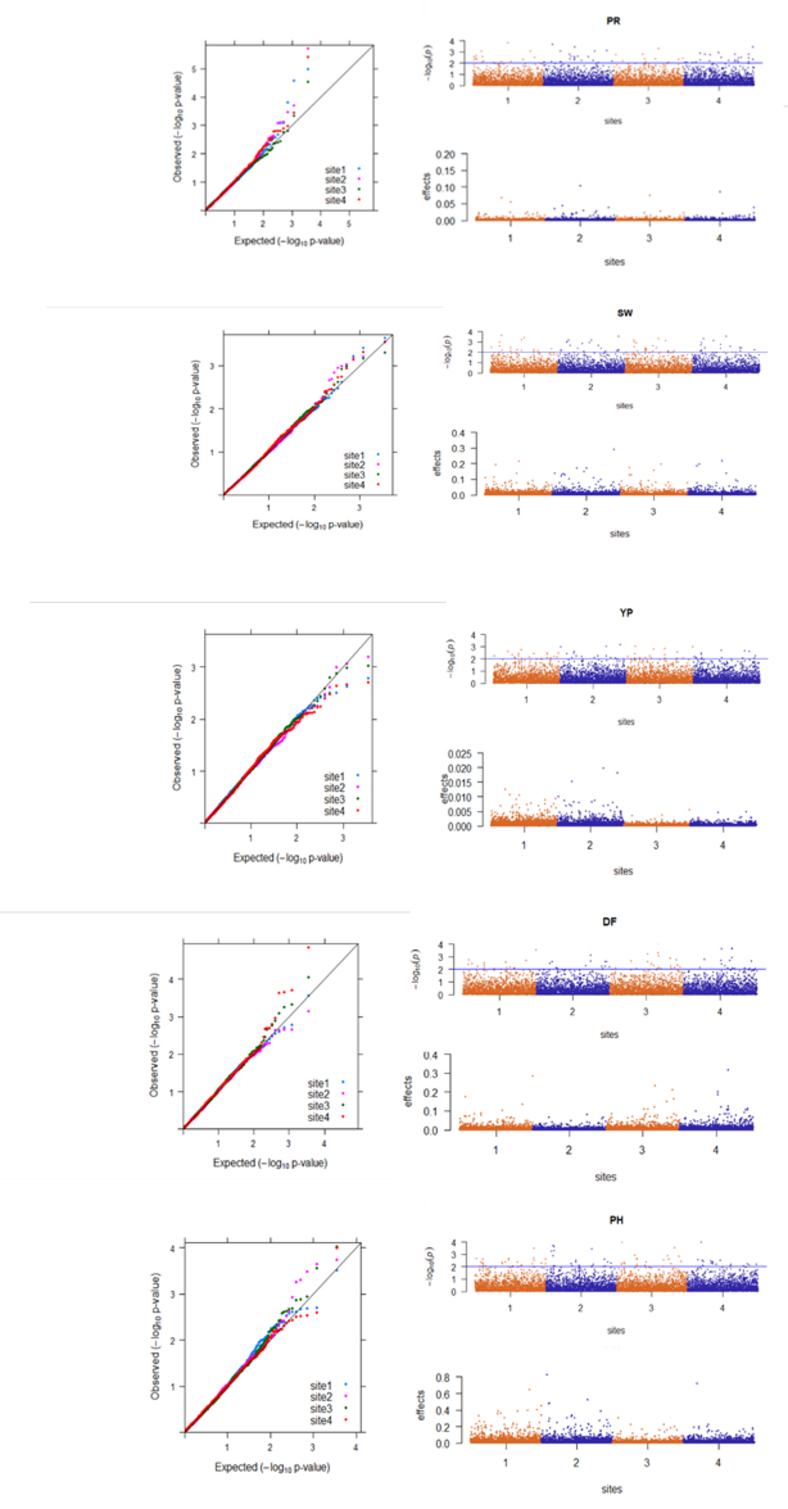


**Supplementary Fig s4**. Heatmap plot of pairwise LD between significant associated SNPs for each trait. Darker colour indicates higher LD between SNPs.


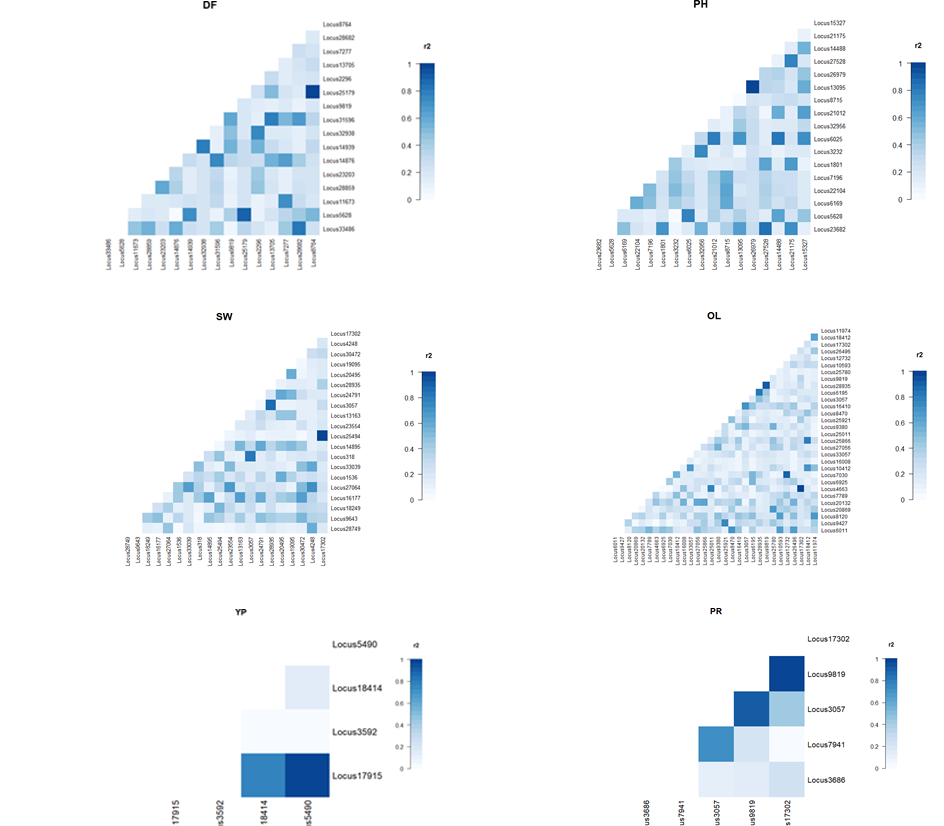

Supplement: Supplementary file 1 — Supplementary file1 (DOCX 973 KB) [file 11032_2022_1295_MOESM1_ESM.docx]
